# Development of Test Equipment for Pedestrian-Automatic Emergency Braking Based on C-NCAP (2018)

**DOI:** 10.3390/s20216206

**Published:** 2020-10-30

**Authors:** Zhiqiang Song, Libo Cao, Clifford C. Chou

**Affiliations:** 1 State Key Laboratory of Advanced Design and Manufacturing for Vehicle Body, Hunan University, Changsha 410082, China; szqhd@hnu.edu.cn; 2 College of Agricultural Engineering, Jiangsu University, Zhenjiang 212013, China; 3 Biomechanics Research Centre, Wayne State University, 818 W. Hancock, Detroit, MI 48201, USA; ccchou@wayne.edu

**Keywords:** pedestrian-AEB, C-NCAP, automated driving robot, GPS differential positioning, test and evaluate

## Abstract

In order to evaluate the effectiveness of a pedestrian-automatic emergency braking (PAEB) system on pedestrian protection, a set of PAEB test equipment was developed according to the test requirement of China-New Car Assessment Program (C-NCAP) (2018) in this study. In the aspect of system control strategy, global positioning system (GPS) differential positioning was used to achieve the required measurement and positioning accuracy, the collaborative control between the PAEB test equipment and automated driving robot (ADR) was achieved by wireless communication, and the motion state of the dummy target in the PAEB system was controlled by using the S-shaped-curve velocity control method. Part of the simulations and field tests were conducted according to the scenario requirements specified in C-NCAP (2018). The experimental and simulated results showed that the test equipment demonstrated high accuracy and precision in the process of testing, the dummy target movement was smooth and stable, complying with the requirements of PAEB tests set forth in C-NCAP (2018), and yielding satisfactory results as designed. Subsequently, the performance of the AEB of a vehicle under test (VUT) was conducted and the score for star-rating to evaluate the performance level of AEB calculated. Results indicated the developed test equipment in this study could be used to evaluate the performance of the PAEB system with effectiveness.

## 1. Introduction

According to the World Health Organization (WHO), today, road traffic injuries are the leading killer of people aged 5–29 years, and 54% of them are pedestrians, cyclists, and motorcyclists [1,2]. With vast availability of sidewalks and bike routes (or paths), and the public encouragement of a modal shift towards cycling and walking, the popularity of pedestrians and cyclists is rapidly growing in the world. As a consequence, an increasing number of car-to-pedestrian or car-to-cyclist accidents is rising as well, thus spurring more attentive actions for the protection of vulnerable road users (VRUs). In the USA, there were 4699 pedestrian fatalities in 2007, but this increased to 5987 in 2016, i.e., by 27.7% [3]. According to the German road safety council, the fault of the drivers mainly leads to the approximately 34,000 accidents involving cars and pedestrians each year [4]. In order to prevent and reduce the severity of pedestrians’ injuries and/or fatalities on roads, car makers and institutes worldwide have been developing preventive means for pedestrian protection using currently available active safety systems (ASS) [5]. The implementation of vehicle crashworthiness tests for preventive pedestrian safety systems and developing realistically associated simulation methods has become a high-priority and urgent challenging task in the New Car Assessment Program (NCAP). To this end, AEB (automatic emergency braking) systems are designed to avoid collisions in the first place or mitigate the severity of impacts if a collision is unavoidable when detecting other vehicles and pedestrians by using sensor technologies and recognition algorithms [6,7,8].

AEB systems can automatically apply the brakes when a VRU is detected while the driver does not respond in time to avoid or mitigate a collision, thus saving countless lives, reducing injuries and societal cost [9]. As such, the AEB system has become a key safety feature implemented into current passenger vehicles, and spread further into the mass market in the future. Though the AEB system has played a key role in reducing traffic accidents, there have been many studies to enhance its performance. However, many car-to-pedestrian crashes occur when a pedestrian is crossing street in front of vehicles. As pedestrians are composed of a wide variety of sizes, move in all directions, and wear clothes made of different clothing materials, their appearances constitute more variables than cars to AEB systems. It is critically to ensure that such PAEB systems are capable of performing adequately in a wide range of scenarios to offer a potential reduction of VRUs fatalities and injuries [10]. An AEB system consists of perception level, decision-making level, and execution level subsystems. Each subsystem is developed by different vendors (or suppliers) with different types of sensors and different control algorithms. How and when AEB systems will be activated depend on control algorithms developed by original equipment manufacturers (OEMs) and suppliers. Therefore, considerably varying performances exist among different AEB systems currently available.

In addition, how effective is the AEB system on avoiding/mitigating rear-end impacts in real-world traffic may be a “sale” safety feature for consumers when making a purchase decision. Up to now, there are no standardized technical requirements for evaluating the effective performance of PAEB systems. In order to improve the performance of PAEB, there are concerns and issues that need to be collectively addressed by regulators, safety researchers, OEMs, suppliers, and others. Many countries have published their own NCAPs (New Car Assessment Programs) to evaluate the effective performance of PAEB systems on protecting VRUs, but the test protocols are not exactly the same among different NCAPs. The National Highway Transportation Safety Administration (NHTSA) has issued a directive of the US-NCAP, including new PAEB testing apparatuses and test procedures to evaluate its benefits [11]. European New Car Assessment Program (Euro-NCAP) is the leading NCAP in the world to address pedestrian protection [12], and Euro-NCAP published a roadmap document for the timeframe 2020 to 2022 [13], which proposed several requirements for VRUs protection such as cross-junction AEB systems and steering intervention. Soon after, China New Car Assessment Program (C-NCAP) was established in reference to the Euro-NCAP as a Chinese counterpart safety rating system. There are some differences in test scenarios (including the speed and deceleration of VUTs) and rating systems specified among C-NCAP, Euro-NCAP, and other NCAPs. It should be noted that NCAPs do not specify what test equipment should be used.

To perform a reliable test for evaluation of the PAEB systems installed in a VUT (vehicle under test) for pedestrian protection, a most feasible and relevant test scenario associated with test protocol, target and proving ground needs to be studied and established. Highly accurate and reproducible test equipment also needs to be developed for the evaluation and verification of their performance on field tests. In Europe, PROSPECT, proactive safety for pedestrians and cyclists, is a collaborative research project focusing on VRUs (vulnerable road users) protection. Thus far, most of the deliverable outcomes, including test cases, study of acceptance, and test protocol, were reported and/or presented without revealing any detailed test equipment information [14,15,16,17,18]. Moreover, these existing papers did not reveal in-depth information about their control systems and control methods because of confidentiality. Therefore, the objective of this study is to develop a set of PAEB test equipment for the evaluation of the performance of a PAEB system based on requirements as specified in C-NCAP (2018 version).

## 2. Related Work

Upon review of relevant information in the literature, it is found that many publications and studies are focused on test scenarios and procedures related the performance of AEB, and those researches pertaining to the PAEB test equipment are scarce. A summary of the related research is as follows.

### 2.1. Test Rig

Messring Company developed an innovative motion rig for pedestrian test, and literature [19] developed a 6-DOF (degrees of freedom) pedestrian dummy to simulate human movement patterns, but they only disclosed the design description of the product, without providing in-depth information and/or study of its control system and control method because it is of a proprietary nature. Zindler et al. [20], and Hahn et al. [21] described a method developed for the PAEB test equipment, without presenting the construction of the test equipment. Sandner [22] developed a balloon target to assess different AEB systems and showed the performance of this balloon target to the consumers. Blank et al. [23] developed a test rig with gantry of poor portability for positioning a pedestrian dummy in driving tests, without disclosing the development of pedestrian and the algorism of cooperative control system. Dummy targets developed from 4 active Systems Company in Austria having higher degrees of freedom to demonstrate multiple poses for injury risk assessment that can be used [17]. The existing driving robots developed based on requirements specified in Euro-NCAP by AB Dynamics Company in the United Kingdom are used by many NCAPs test laboratories worldwide [24].

### 2.2. Cooperative Control Systems

Aparicio et al. [25], Ambos [26], and Fritz et al. [27] proposed the cooperative control systems for communication between each other’s position and velocity between the VUT and pedestrian dummy target by WLAN without description of their cooperative control systems. Moreover, it is important to predict error with machine learning methods at a local level, and with artificial intelligence for decision-making at a global level in the test equipment [28,29].

### 2.3. Test Procedures

Lenard et al. [30] reported the development of AEB test procedures based on empirical data from STATS19 [31] and from the in-depth on-the-spot (OTS) [32]. Duan et al. [33] analyzed the driver’s braking behavior in vehicle-bicycle conflicts in China, and used results to improve the test protocol enactment of bicyclist-AEB systems. However, technical information pertaining to their test equipment, control systems, and control methods remain undisclosed because of their proprietary nature. Olaf et al. [34] proposed test scenarios in a CATS (Cyclist-AEB testing system) project, and provided the proof of test scenarios and the feasibility of practical implementation of such scenarios in Euro-NCAP for testing.

### 2.4. The Performance of AEB Systems

Park et al. [35] proposed pedestrian target selection using a funnel map for a PAEB system to determine the precise warning and brake timing. Lenard et al. [36] studied the position of pedestrians and pedal cyclists relative to the vehicle with AEB before impact, and considered the information to be essential for the development of effective AEB systems and relevant test conditions for consumer ratings. Zhang et al. [37] proposed a hierarchical control structure of an AEB system based on Nonlinear Model Predictive Algorithm to reduce collision risk and improve driving comfort. Rosén [38] studied how the performance of an AEB influenced real-life effectiveness, which was measured in terms of the reduction of fatalities and severe injuries of AIS3+F.

Based on the aforementioned reviews, the relevant published literature did not provide in-depth information regarding associated control system and control methods as well because of its proprietary nature, and the existing commercial equipment was developed based on other NCAPs. Moreover, the test protocols specified in C-NCAP (2018 version) are not exactly the same as those specified in other NCAPs (see Discussion section for examples), so the control variables (including relative distance, test speed, and deceleration) in the control algorithm will be different. Therefore, it is necessary to develop a set of test equipment based on requirements specified in C-NCAP (2018 version) in this study. The main objective of this paper is to develop the first set of test equipment based on requirements specified in C-NCAP (2018) in attempt to make some contribution to the body of knowledge in active safety field. This paper not only disclosed the design description of the subsystems of test equipment for a PAEB to be used in C-NCAP (2018 version), but also studied its control strategy and control method. Especially, this paper adopted the measurement methods of global positioning system real-time kinematic (GPS-RTK) positioning and a five-stage S-shaped acceleration and deceleration control method for controlling velocity of the dummy target’s motion to improve the test accuracy and precision of the developed test equipment.

Following the introduction given in Section 1 and related work in Section 2, Section 3 presents analysis of the assessment protocol of PAEB specified in C-NCAP (2018). Section 4 outlines the research methodology. In Section 5, the development and design of the components of PAEB test equipment are described, and cooperative control strategy used in the PAEB test equipment is studied. Section 6 presents field tests to verify the feasibility and accuracy of the developed PAEB test equipment. Section 7 uses the developed AEB test equipment to test and evaluate the PAEB system installed in a VUT, and calculates the score of the PAEB system based on the methods specified in C-NCAP (2018). Section 8 provides the main research results. The contributions of this study to the active safety field and research methods are discussed in Section 9. Finally, conclusions are drawn, and future research studies are outlined in Section 10.

## 3. Analysis of the Assessment Protocol of PAEB Specified in C-NCAP (2018)

### 3.1. PAEB Test Scenarios Specified in C-NCAP (2018)

**Figure 1:**
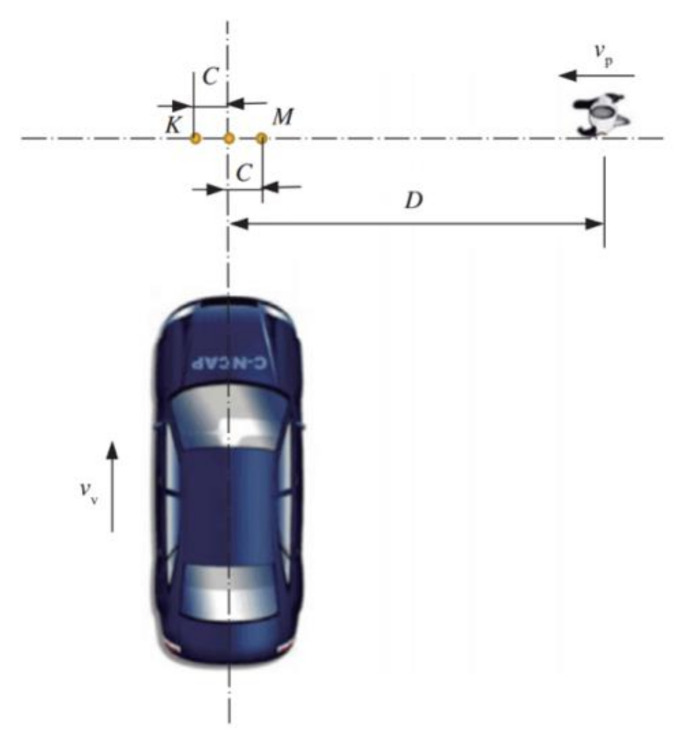
The sketch of CVFA-50 test scenario.

For PAEB experiments, there are four test scenarios as specified in C-NCAP (2018) [39], including (a) evaluation of car approaching to VRU adults who go from far-side cross, and the collision point is at 50% of the car width (*L* point, which is located between points K and M and not shown in Figure 1), and this case is denoted as car-to-VRU Far-side Adult-50, which is abbreviated as CVFA-50. (b) car-to-VRU Far-side Adult-25 (CVFA-25), (c) car-to-VRU Near-side Adult-25 (CVNA-25), and (d) car-to-VRU Near-side Adult-75 (CVNA-75). Figure 1 depicts the schematic of the CVFA-50 test scenario, namely a car approaches a pedestrian crossing a street in the longitudinal direction, and crashes into him at an impact location about 50% of the car width.

Referring to Figure 1, D is the required travel distance of the pedestrian dummy target, and is different depending on required test scenarios; for example, D is set for 6 m in far-side test scenario, or 4 m in near-side test scenario. Point M is at the 25% of the car width, while Point K is at the 75% of the car width. The reference point is the starting point of the dummy target. The speed of the dummy target, *v_p_*, is 5 km/h in near-side test scenario or 6.5 km/h in far-side test scenario, while the speed of the vehicle under test (VUT), *v_v_*, is set at 20, 30, 40, 50, and 60 km/h, respectively. During these tests, the speed accuracy requirements of the VUT and the dummy target controlled by control systems are within ±0.5 km/h, and ±0.2 km/h, respectively, and the measurement accuracy of the speed is within ±0.01 km/h. To ensure the speed accuracy of VUT and pedestrian dummy target, an automated driving robot (ADR) installed in VUT should be used to automatically operate the VUT. An S-shaped curve for controlling velocity should be used to improve the speed accuracy of the dummy target’s motion. In addition, the VUT and the dummy target should be equipped with a global positioning system (GPS) and wireless communication module, respectively.

### 3.2. The Scoring Standards of Vehicle Safety in the C-NCAP (2018)

**Figure 2:**
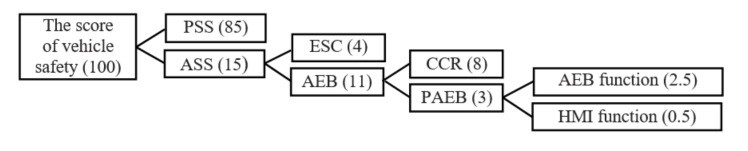
The relationship of scoring hierarchy.

Analysis of the scoring standards of vehicle safety in the C-NCAP (2018) is helpful to score the performance of PAEB system. Figure 2 shows the relationship of scoring hierarchy specified in C-NCAP (2018). The total score of passive safety system (PSS) is eighty-five (85) points, the total score of active safety system (ASS) is fifteen (15) points, including eleven (11) points of AEB and four (4) points of ESC (electronic stability control). The scoring standards in AEB system is composed of eight (8) points for car-to-car rear collision (CCR) (including the leading car stationary, moving at low speed, and braking at constant deceleration, namely CCRs, CCRm, CCRb, (see abbreviation at the end of this paper), respectively) and three (3) points for PAEB. The PAEB scoring system consists of AEB function and human–machine interface (HMI) using weighted values of 5 and 1, respectively.

### 3.3. Method of Calculating Score of PAEB Systems

The performance of the AEB systems installed in a VTU is evaluated by calculating its score based on reduction of the VUT’s velocity. The method of calculating score for an AEB system specified in C-NCAP (2018) is described below: (a) When the test speed of the VUT is less than 40 km/h, and if the VUT can brake to stop before hitting the dummy target to avoid collision, a full score is granted at the test speed point. In case the VUT cannot brake to stop before hitting the dummy target, the score at this test speed is calculated based on percentage of reduction of relative velocity, namely the scoring rate of test speed = (*v_test_* − *v_impact_*)/*v_test_*, where *v_test_* is the initial VUT velocity and *v_impact_* is the velocity at collision. (b) When the test speed of the VUT is higher than 40 km/h, and if the AEB function reduces the speed of the VUT more than 20 km/h, the AEB gets the full score at this test speed. Otherwise, the AEB gets the zero score at this test speed.

### 3.4. Method of Calculating Score for Human Machine Interface (HMI)

The functions of AEB and forward collision warning (FCW) are in the state of “on” by default is a prerequisite of HMI score when the vehicle is starting. There are two scoring items: (a) The AEB and FCW functions cannot be closed by a single key operation. (b) When the speed of the VUT is higher than 40 km/h, a pedestrian is detected, and the FCW alarm system should give a loud and clear warning to the driver when TTC (time to collision) is equal to 1.2 s. Both of the two items are prerequisites for evaluating the scores of HMI.

## 4. Methodology

The methodology used in this study consists of the following tasks:

(1) Develop and design components of the PAEB test equipment including a dummy target and its traction system, an automated driving robot with a monitoring device for warning performance, and associated data operating control software. The equipment and associated subsystems will be presented in Section 5.1.

(2) Propose a cooperative control system along with control algorithm for conducting PAEB test scenarios specified in C-NCAP (2018). Formulate cooperative control strategy for the PAEB test equipment, including the cooperative control for dummy traction system and VUT, control algorithm for dummy’s motion, and calculation of the target points in cooperative control. Based on the aforementioned strategies, perform simulation of the test scenarios as specified in C-NCAP (2018) using MATLAB/Simulink for verification. This task will be presented in Section 5.2, Section 5.3 and Section 5.4.

(3) Conduct field tests using the developed PAEB test equipment including testing the accuracy of the speed and movement distance of the dummy target by using different SUVs with AEB systems, testing the effectiveness of cooperative control between dummy target traction system and automated driving robot. Details of this task will be presented in Section 6.

(4) Using the developed test equipment to test the performance of the PAEB installed in a vehicle, compare these test results with those assessed by the China Automotive Technology and Research Center (CATARC), which will be presented in Section 7.

## 5. Development of a PAEB Test Equipment and Analysis of the Control Strategy

### 5.1. Development of Components of the PAEB Test Equipment

In order to reproduce the test scenarios, a set of test equipment is developed. This test equipment mainly includes a dummy target which is used as a surrogate to pedestrian, a dummy target traction system, an automatic driving robot (ADR), a monitoring device for warning sound, and a data operating control platform real-time communication module. General requirements of those mentioned above are being weatherproof and corrosion resistant, easy to assemble and disassemble, and optimized-in-weight for portable use in outdoor environments. More importantly, as the test equipment is designed for extended use to evaluate the performance of AEB, therefore, the reproducibility and accuracy of the test equipment is highly demanded. Each of these subsystems listed above are developed and described in subsequent subsections.

#### 5.1.1. Development of Dummy Target

**Figure 3:**
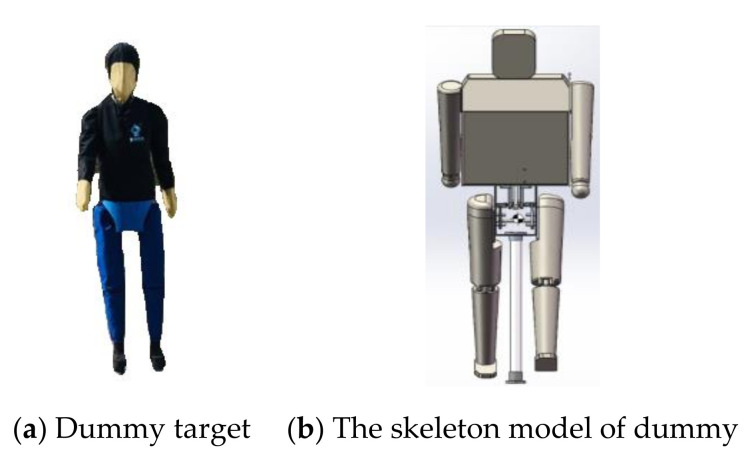
Dummy target for simulating pedestrian.

The dummy target must have an appearance of a real human with three-dimensional shape to mimic human beings, namely pedestrians. In addition, it needs to have the feature of radar response, infrared reflectivity, visual appearance, and lightweight structures [40]. For accurate detection, the dummy target should have pedestrian walking postures. Moreover, the dummy target needs to have a soft durable clothing outside to avoid damaging the VUT when they have a collision. In this study, the dummy target’s bodies are made of foam rubber with their exposed parts being joined by flexible rubber to make it crashworthy. In a test scenario, the dummy target must be programmed to move on a predefined trajectory. When the dummy target is collided with the VUT, it can be dropped from the bracket, on which the dummy target stands, to avoid mutual damage caused by the collision. Both the adult and child dummy targets are developed according to their respective sizes as specified in C-NCAP. They are light, weighing less than 1.5 kg. The standing heights of the adult and child dummy targets are 180 cm and 150 cm, respectively. To develop a dummy target like a real person, the infrared reflective material is applied on dummy’s clothes to improve radar recognition rate under daylight and night-time conditions. The dummy target’s legs are designed to swing as driven by a motion mechanism to simulate proper pedestrian walking posture. This is a very important aspect, because the legs swing generates a micro-Doppler signal to provide additional speed information to enable the AEB to recognize the dummy target as a pedestrian [41]. The adult dummy target and its skeleton model are shown in Figure 3a,b, respectively.

#### 5.1.2. Development of Dummy’s Traction System

**Figure 4:**
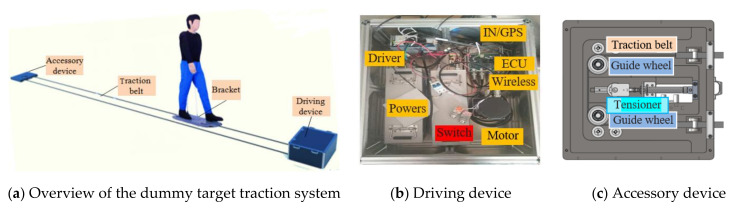
Dummy target traction system.

When the dummy target is programmed to move on a predefined trajectory, a traction system with belt-driven platforms is used to pull it into motion at a given speed. This dummy traction system includes an accessory device, traction belt, bracket, and driving device, as shown in Figure 4a. The driving device includes a wireless communication module, a controller, a motor, and power supply, as shown in Figure 4b. The wireless communication module receives the information of the VUT’s driving speed and position, which are then transmitted to the controller. The controller, in turn, controls the motor to drive the traction belt pulling the dummy target “walk” forward. The dummy target driving controller is composed of a master control chip (STM32F767) and a slave control chip (STM32F103). The master control chip receives the information of the VUT’s velocity and position transmitted by each module, then analyzes, and processes to decide whether or not to start triggering dummy target movement. The slave control chip is responsible for the accurate control of acceleration/deceleration of the driving motor. The accessory device shown in Figure 4c is used to change the direction of the traction belt movement and form a complete circuit. This subsystem is only used to meet the current assessment protocol in the development of autonomous driving technology. However, more advanced pedestrian simulation systems need to be developed to replicate more complex traffic scenarios involving numerous road users.

#### 5.1.3. Development of the Automated Driving Robot

**Figure 5:**
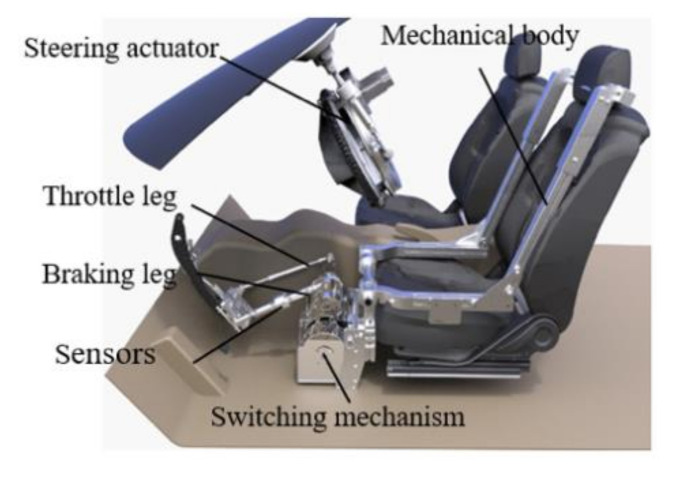
The developed automated driving robot.

An automated driving robot (ADR) is developed to replace the human driver to drive the VUT during the tests. It can accurately control the driving speed and heading angle of the VUT according to the pre-determined test scenarios without the influence of human factors, thus ensuring the test results meet the accuracy requirements as specified in C-NCAP (2018 version). Figure 5 shows the developed ADR, consisting of electronic control units (ECU), a mechanical body, driving motors, actuators (including throttle leg, braking leg, and steering actuator), pressure and displacement sensors, inertial navigation system (INS), and GPS-RTK positioning module.

Mechanical body is used to join throttle leg and braking leg, which can be adjusted to their respectively appropriate positions according to the size of the space available in the car and then be fixed to the driver’s seat. The switching mechanism works as an on/off switch for acceleration operation or braking operation following the instructions from the ECU of ADR. During the test process, the ECU obtains the information of the VUT’s state variables (including acceleration, speed, position, and yaw angle) in real time from the INS and GPS-RTK positioning module installed in the control system, and compares these state variable parameters with the set ones according to the test scenario, then sends corresponding control instructions to the throttle leg or braking leg, and/or steering actuator to adjust the speed and heading angle of the VUT.

#### 5.1.4. Development of Monitoring Device for Warning Performance

**Figure 6:**
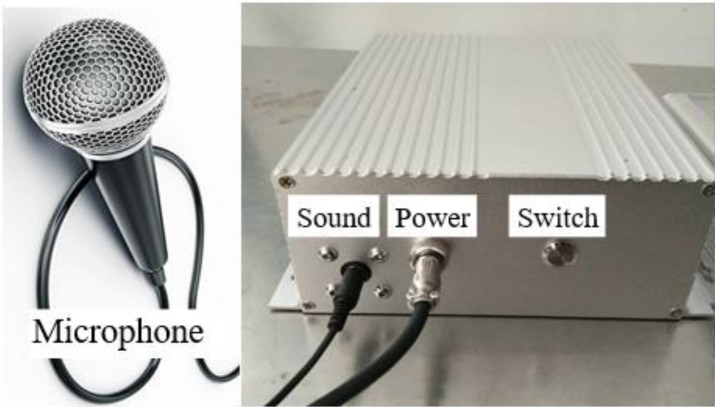
Monitoring device for warning.

In order to check when the warning function of the PAEB system is activated to give a loud and clear alarm to the driver before the VUT impacts the dummy target, a monitoring device for testing the warning performance is developed. This monitoring device consists of a microphone and a signal processing unit, as shown in Figure 6. The microphone is used to collect the warning sound from the warning system of the PAEB system, and the warning sound is transmitted into the signal processing unit. Since time to collision (TTC) is the judgment index for collision warning, the signal processing unit analyzes and calculates the value of TTC according to the time of the collected warning sound, and then displays the value of TTC for evaluating the warning performance of the PAEB system.

#### 5.1.5. The Data Operating Control Software

To record and display the test data, a data operating control software is developed using Microsoft Foundation Classes (MFC) in Microsoft Visual Studio 2017. The data operating control software is used to set the input parameters of the VUT’s initial speed and the dummy target’s motion speed prior to a PAEB experiment according to different test scenarios per C-NCAP. Moreover, the data operating control software has not only the function of setting the input data, but also the function of recording the test data during a test process and displaying the curve changes of position, velocity, acceleration, and heading vector, etc. The data operating control software can record and display test date and test time, sensors perception data, battery status data, and GPS positioning data. The data operating control software reads these data from every sensor and device through the serial port communication. When the data operating control software reads the GPS data from GPS receiver, it needs to follow the protocol of National Marine Electronics Association (NMEA)-0183. Usually, the positioning longitude, positioning latitude, speed, time, and other information can be read from the statement of $GPRMC in NMEA-0183 protocol.

### 5.2. Cooperative Control Strategy for the PAEB Test Equipment

#### 5.2.1. The Method of Cooperative Control for Dummy Target Traction System and ADR

**Figure 7:**
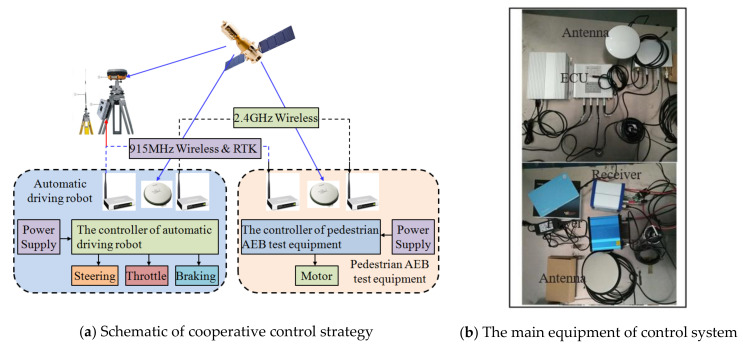
The overall cooperative control for dummy target traction system and ADR.

Figure 7a depicts the overall design of the cooperative control between the dummy target traction system and the ADR. Figure 7b shows key components of the control system such as navigation positioning receiver, positioning data link (PDL), ECU, move power supply, antenna, etc. The dummy target traction system is tested, working synergistically with the ADR, INS/GPS navigation, and positioning module to send real-time position and velocity information to the control system of dummy target traction system wirelessly. The main controller in dummy target traction system triggers the dummy target to start moving when the calculated distance between the VUT and the dummy target traction system is equal to the pre-set value, depending on the requirements of the test scenario for the VUT driving speed, dummy target moving speed, and collision position. Therefore, the main controller has the function to ensure the dummy target arrives at the predetermined position (i.e., impact or collision location).

**Figure 8:**
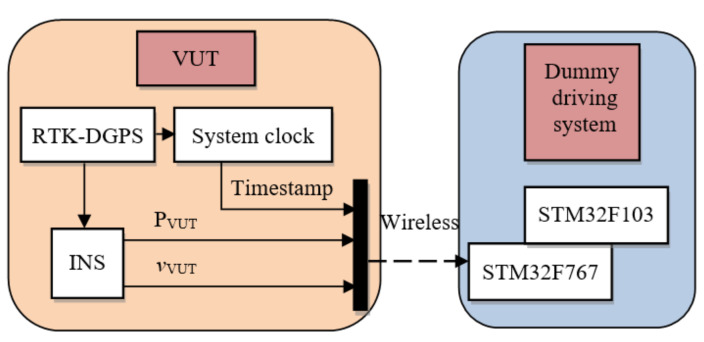
The position and velocity of VUT is measured and transmitted.

As mentioned earlier, the VUT is equipped with ADR, INS, and GPS positioning equipment. The inertial navigator can collect the test speed, acceleration and heading angle of the VUT in real time. The differential global positioning system (DGPS) consists of the VUT positioning, RTK base station positioning and dummy driving system positioning. The DGPS can obtain centimeter-level positioning accuracy, thus achieving high-precision positioning of both the VUT and dummy target motion [42]. ADR can control the VUT according to the vehicle trajectory set in the intended test scenario. The real-time VUT’s position and velocity are measured by an integrated navigation system of RTK-DGPS/INS installed in the VUT and transmitted to the controller of the dummy traction system (see Figure 8) via 2.4 GHz wireless communication channel.

Differential precise positioning information is communicated via 915 MHz wireless communication channel, while the information of vehicle distance and velocity is communicated through the 2.4 GHz wireless communication channel (NRF24L01 chip developed by Nordic Corporation). The controller of dummy traction system monitors the VUT whether or not traveling to the predefined location. The coordinates of the predefined location are calculated by the controller of dummy target driving system depending on the currently undertaken test scenario (the requirements of the test scenario for the driving speed of the VUT, the moving speed of the dummy target and the collision position of the VUT). When the VUT arrives at the predefined position, the controller immediately starts the dummy target to move and controls the velocity of dummy target motion to follow the S-shaped path to be described in Section 5.2.2. Finally, the contact between the VUT and the dummy target at the pre-determined collision point is realized.

#### 5.2.2. Control Algorithm for Dummy’s Motion

During the testing process, the dummy target needs to move from the original starting position to the targeted one, thus requiring to start, accelerate, move steadily, and then slow down until stopping at the targeted location. In order to keep steady motion of the driving motor and dummy target in this process, the motion control method of S-shaped curve of acceleration and deceleration is adapted in this study. The shape of the velocity curve is S-shaped in the acceleration and deceleration stages, with both acceleration and velocity curves being continuous, and the derivative of acceleration (i.e., jerk = da/dt) being constant. By controlling the jerk value, the impact on the dummy target motion can be minimized, thus leading to realization of the steady motion of the dummy target, and achieving the flexible acceleration and deceleration control. This control strategy provides advantages of good stability, small jerk force, and high positioning accuracy to the dummy target motion. In addition, this control strategy also has extremely high practical application merits, especially use of the velocity control method incorporating a very important technology capable of dealing with modern high-speed and high-precision machine [43].

**Figure 9:**
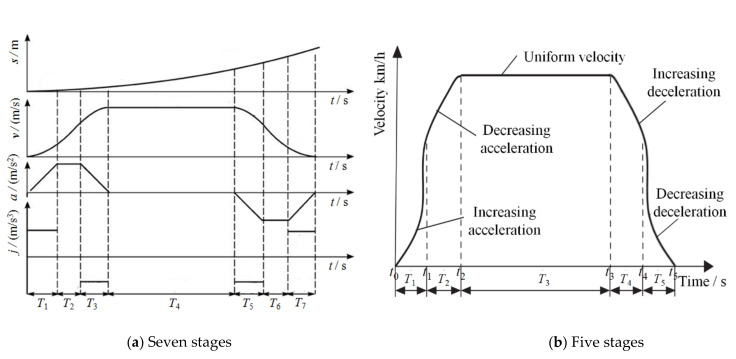
S-shaped velocity curve with acceleration and deceleration.

Generally, the acceleration and deceleration motion of the S-shaped curve, including seven or five stages of motion control, is described as follows [44]:

(1) When the maximum acceleration of the control system cannot reach the required speed, a uniform acceleration stage is needed. Should this occur, the movement process of the S-shaped acceleration and deceleration curve is then divided into seven stages: increasing acceleration stage, uniform acceleration stage, decreasing acceleration stage, uniform velocity stage, increasing deceleration stage, uniform acceleration stage, and decreasing deceleration stage, as shown in Figure 9a.

(2) When the maximum acceleration of the control system is able to reach the required speed, there is no need to have a uniform acceleration stage, namely, *T*_2_ = *T*_6_ = 0 in Figure 9a. Hence, in this case, there are only five stages in the S-shaped velocity curve to accommodate with acceleration and deceleration motion, namely, increasing acceleration, decreasing acceleration, uniform speed, increasing deceleration, and decreasing deceleration, as shown in Figure 9b. Let *t_k_* (*k* = 0, 1,…, 5) represent the starting time of each stage, then *T_k_* = *t_k_* − *t_k_*_−1_ (*k* = 0, 1,…, 5) represents the running time of each stage.

In this study, a five-stage S-shaped acceleration and deceleration control method is selected because of its simple control algorithm, since the dummy target’s velocity is not high, and the driving motors can easily reach this target velocity. In practice, the dummy target’s starting and ending velocities are zero. In the acceleration and deceleration stages, the absolute value of *j* is taken to be a fixed value, so that the acceleration curve becomes a triangle in the starting and the stopping stages. In order to make the acceleration and deceleration process symmetrical, the time of increasing acceleration stage is equal to that of decreasing acceleration stage, and the time of increasing deceleration stage is equal to that of decreasing deceleration stage, namely *T*_1_ = *T*_2_ and *T*_4_ = *T*_5_. Since *T*_1_ = *T*_5_ and *T*_2_ = *T*_4_, and letting *T*_1_ = *T*_2_ = *T*_4_ = *T*_5_ = *T* (see Figure 9b), then, as long as *T*_1_ and *T*_3_ are determined, the formulas for calculating acceleration *a* and velocity *v* of the dummy target can be derived accordingly.

In the acceleration and deceleration stages, jerk is the first derivative of acceleration, and the relationship between jerk and acceleration is expressed in Equation (1), and the relationship between velocity and acceleration is given in Equation (2).

(1) $$j(t)=\frac{d\left[a(t)\right]}{dt}$$

(2) $$a(t)=\frac{d\left[v(t)\right]}{dt}$$

Due to the movement speeds of the dummy target in different test scenarios are specified in C-NCAP (2018), then let one assume that the targeted velocity of the dummy target is *v_t_*. The absolute value of jerk is set to be a constant value *j*, and the jerks at time *t*_0_, *t*_2_ and *t*_5_ (see Figure 9b) are set to be zero, then the acceleration of the dummy target reaches its maximum value at time *t*_1_ in the five-stage S-shaped curve model, the maximum acceleration *a_max_*, and velocity at time *t*_1_ are expressed as Equations (3) and (4).

(3) $$a_{\max}=jt_{1}$$

(4) $$v(t_{1})=\frac{1}{2}jt_{1}^{2}$$

In the five-stage S-shaped curve model, *T*_1_ = *T*_2_ = *T*_4_ = *T*_5_ = *T*, and *v*(*t*_0_) = *v*(*t*_5_) = 0 (see Figure 9b), the targeted velocity *v_t_* is expressed as:

(5) $$v_{t}=2\times v(t_{1})=jt_{1}^{2}$$

The duration from time *t*_0_ to time *t*_1_ is *T*_1_ (see Figure 9b), and *T*_1_ = *T*_2_ = *T*_4_ = *T*_5_ = *T*, then the duration *T_i_* (*i* = 1, 2, 4, 5. Please note that 3 is not included here) is calculated according to Equation (6).

(6) $$T_{i}=\sqrt{\frac{v_{m}}{j}}=\frac{a_{m}}{j}$$

According to the above theoretical mathematical models, the acceleration, velocity, and displacement of each stage in the S-shaped curve can be calculated as long as the targeted velocity *v_t_* and duration *T* are determined.

**Figure 10:**
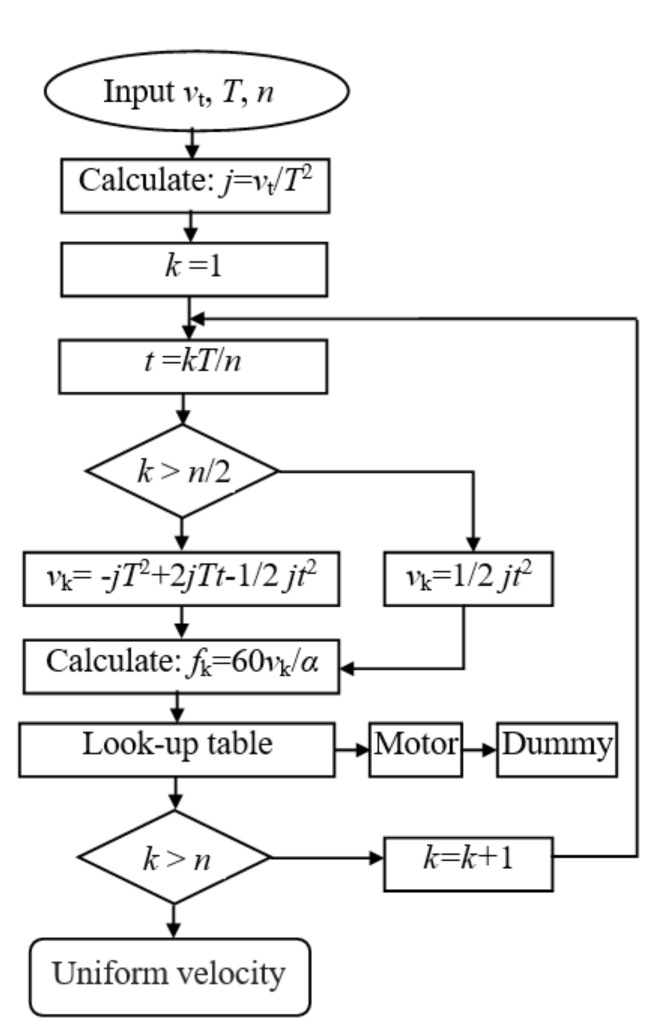
The flow chart of the program of increasing velocity stage in the S-shaped velocity curve.

After calculating the running time of five stages in the S-curve acceleration and deceleration process, the flexible acceleration and deceleration control algorithm can be easily implemented into the motion controller based on STM32F103 to improve the motion performance of the dummy target and the function of the whole system. The flow chart of the program of increasing velocity stage in the S-shaped velocity curve is shown in Figure 10. In this flow chart, *v_t_* is the required velocity of dummy target; *T* is the time of increasing acceleration for motor; *n* is the number of samples per cycle; *α* is the step angle of motor; *f_k_* is the pulse frequency. The section of speed reduction is opposite to that of a speed increase [45].

Initial and final velocities of the dummy target are zero. According to kinematics principles, the expressions of acceleration *a*(*t*), velocity *v*(*t*), and displacement *S*(*t*) of the dummy target in each stage are shown in Table 1.

**Figure 11:**
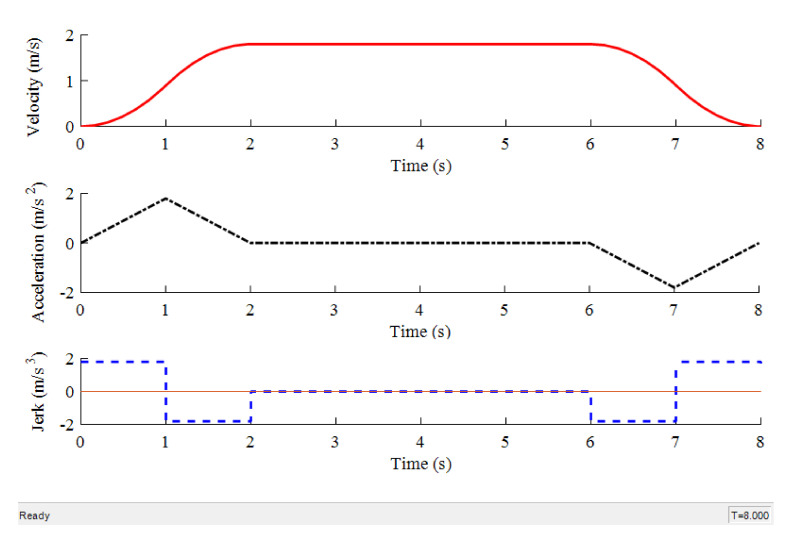
The motion state of dummy.

The required movement speed of dummy target is 6.5 km/h (1.80 m/s) in CVFA-50 test scenario specified in C-NCAP (2018), and *T* is assigned a value of 1 s. Using the expressions $v_{t}=jt_{1}^{2}$ and $a_{m}=jt_{1}$, then *j* is found to be 1.80 m/s^3^ and *a_m_* = 1.80 m/s^2^. The expressions of acceleration, velocity, and displacement for each stage in Table 1 are simulated using MATLAB/Simulink that yields the simulation results as shown in Figure 11.

From the simulation results shown in Figure 11, the motion state curves (velocity, acceleration, and jerk) of the dummy target are obtained using the expressions given in Table 1, which are in accord with the intended characteristics of the S-shaped acceleration and deceleration curve exhibiting that the curves of velocity and acceleration are continuous, and the absolute value of jerk is a constant value. Therefore, the dummy target’s motion will be reliably controlled to be smooth without “jerk” during movement by the S-shaped velocity curve method using acceleration and deceleration control algorithm. At the same time, the expressions given in Table 1 are verified to be correct. The detailed data of the simulation results of the S-shaped velocity curve are shown in Table 2.

### 5.3. Calculating the Position of a Target Points in Cooperative Control

**Figure 12:**
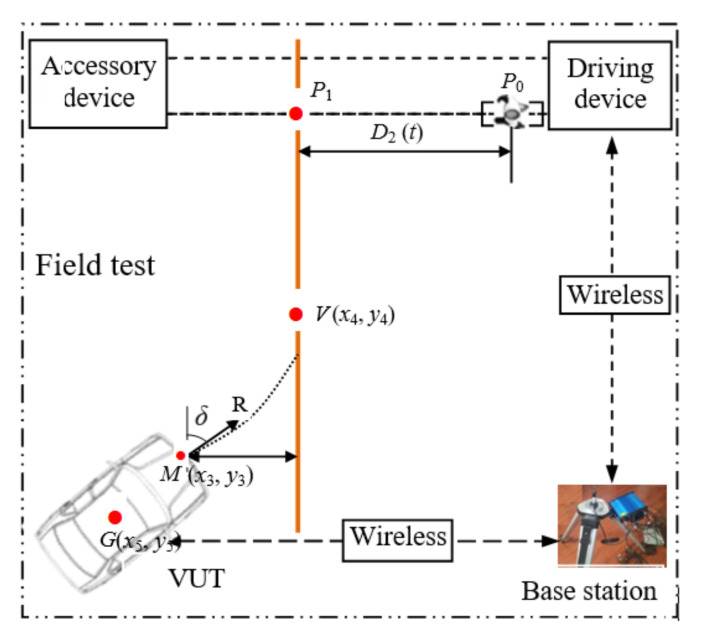
Adjustment of the orientation and position of the VUT for collaborative control.

Before a field test, the position and orientation of the VUT may not be in the desired state for the test, thus the orientation and position of the VUT and the cooperative control system needs to be adjusted prior to a test (see Figure 12).

The coordinates of the midpoint of the width at the front of the VUT *M* (*x*_3_, *y*_3_) and vehicle heading vector *R* are obtained by using the mobile station. The distance (*D*_2_) between the center point *P*_0_ and line *VP*_1_, and the angle (*δ*) between the vehicle heading vector *R* and *VP*_1_ are calculated by the controller of the ADR. By turning the steering wheel, the ADR adjusts the heading angle and position of the VUT in real time until the longitudinal center line of the vehicle coincides with the target trajectory (*VP*_1_). This procedure completes the adjustment of the position and orientation of the VUT.

The coordinates of *P*_0_ (*x*_0_, *y*_0_) (initial position of dummy target) and *P*_1_ (*x*_1_, *y*_1_) (collision point location) are measured using the mobile station of pedestrian detection system. When the dummy target is triggered to start moving depends on the position coordinates of Point *V* (*x*_4_, *y*_4_), where the VUT will arrive. Of course, the position coordinates of Point *V* can be obtained depending on the time (*t*) which is required to take the dummy target to move to the preset collision point and the speed of the VUT, *v*, at that time. Basically, this is the longitudinal distance calculated from *S* = *v* × *t*.

When the VUT is moving, the coordinates of Point *M* (*x*_3_, *y*_3_) keep changing, and are updated in real time by the positioning system of the VUT. The updated coordinates of Point *M* (*x*_3_, *y*_3_) are then sent to the controller of the dummy target traction system through the wireless module in real time. When *x*_3_ = *x*_4_ and *y*_3_ = *y*_4_, namely the coordinates of Point *M* and Point *V* are equal, it is time for the driving controller to trigger the dummy target to start walking at the predetermined speed to Point *P*_1_, where the VUT will be driven to arrive at the same time.

### 5.4. Simulation of the Cooperative Movement in Test Scenarios

In this Section, the cooperative movement between VUT and dummy target in the test scenarios of CVFA-50 is simulated and analyzed using MATLAB/Simulink to verify the control algorithm. In these test scenarios, the dummy target moves a distance of 6 m and the speed is 6.5 km/h (1.8 m/s) in duration *T*_3_ as shown in Figure 9b. Therefore, the displacement of the dummy target before *t*_3_ in the five stages S-shaped velocity curve model is calculated only to meet the test requirements of distance. According to Table 1, the displacement is expressed in Equation (7):

(7) S=S1+S2+S3=16jt13+jt12t

Based on the above analysis of control algorithm for dummy target’s motion in Section 5.2, *t*_1_ = 1.00 s, *j* = 1.80 m/s^3^, *a_m_* = 1.80 m/s^2^. Substituting these values along with *S* = 6 m into Equation (7) yields *t* = 3.17 s. The total walking time of the dummy target is 5.17 s. Based on the total walking time of the dummy target, the longitudinal coordinate values of Point M and the vehicle center Point G at test velocities ranging from 10 km/h to 60 km/h with an increment of 10 km/h are shown in Table 3.

**Figure 13:**
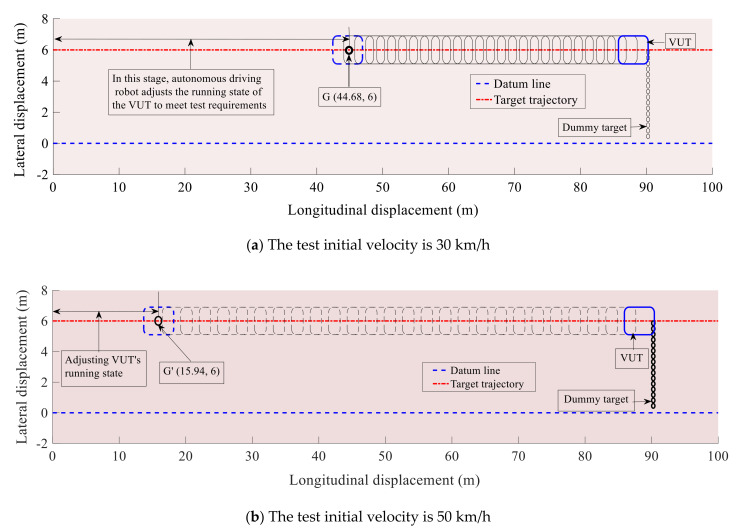
The demonstration process diagrams for the VUT and dummy target.

Figure 13a,b are the demonstration process diagrams in MATLAB/Simulink simulations at the test velocity of 30 km/h and 50 km/h. In these figures, the small black circle, the blue rectangle, and the red straight line represent the dummy target, the VUT, and the predetermined VUT trajectory, respectively.

From Figure 13a,b, it can be seen that both the VUT and the dummy target are able to reach the predetermined collision point at the same time, using the aforementioned control strategy and control algorithm, of which the rationality and feasibility are hence verified. In addition, theoretical results thus obtained can also be used as references for comparison with corresponding experimental results.

## 6. Debug-Testing the Performance of the PAEB Test Equipment in Field Tests

Now, it is at the position to conduct field tests for debugging the PAEB test equipment which is divided into two groups. One group is to test the motion speed of the dummy target, and the other is to test the cooperative control performance of the PAEB test equipment between the dummy traction system and the ADR. In the cooperative control system, the communication technology and control algorithms are the key factors for the cooperative control performance.

### 6.1. Testing Control Accuracy of the Dummy Target Traction System

**Figure 14:**
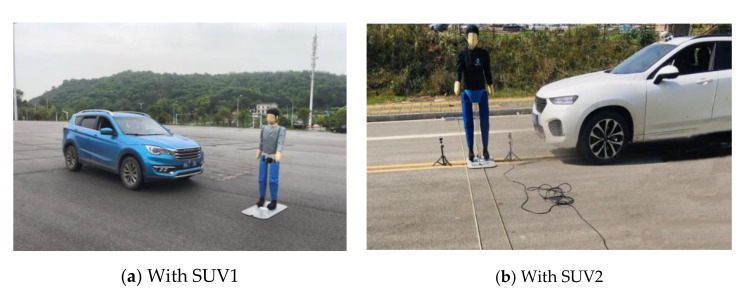
Test the control accuracy of the dummy target traction system for the speed of the dummy target.

To test the control accuracy of the dummy target traction system for the dummy target motion’s speed and distance, two SUVs, designated as SUV1 and SUV2, from different OEMs, whose widths are 1925 mm, and 1931 mm, respectively, are used as VUTs in field experiments in test scenarios defined in the C-NCAP (2018), as shown in Figure 14. The dummy traction system controls the speed of the dummy target motion using the S-shaped acceleration and deceleration control algorithm as discussed above. The standard values and test results of each test group are shown in Table 4 and Table 5, respectively. A remark is made here that the accuracy of data collection and recording for dummy target’s speed is ± 0.01 km/h, the accuracy of data collection and recording for dummy target’s lateral position is ± 0.03 m, and the accuracy of the dummy target’s speed controlled by the dummy target traction system is 0.2 km/h, which are specified in C-NCAP (2018). Therefore, the test results should be within the valid range of each test scenario, as also shown in Table 4, and the test results that failed (i.e., outside the efficient range) in meeting test requirements are displayed with bold-faced underlines.

In a total of 48 tests of the walking distance and speed of dummy target, only distances of two tests in Table 4 fell outside the valid range in the test scenarios of CVFA-50 and CVNA-25, while all the test results in Table 5 on another test site ground fell in the valid range. Reasons for causing such an error are judiciously believed to be due to the uneven friction coefficient on the test site ground and/or the tension of the traction belt. In addition, most of the test results are within the prescribed valid range, and the satisfactory rate of the dummy target traction system in PAEB test equipment for controlling the dummy target walking distance and speed is 95.83%, which is determined by the number of tests fell into the valid range divided by the total number of tests conducted. Therefore, it can be concluded that the PAEB test equipment is capable of achieving accurate control of the dummy target movement in comply with the C-NACP (2018) test requirements based on the satisfactory rate obtained.

### 6.2. Testing Cooperative Control Performance of the PAEB Test Equipment

**Figure 15:**
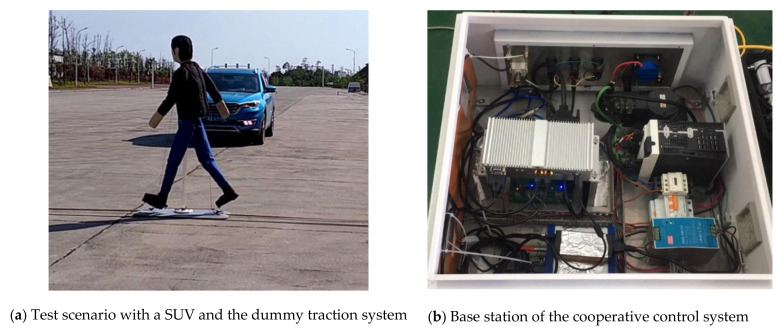
Test cooperative control performance of the PAEB test equipment in field tests.

Cooperative control between the dummy target traction system and ADR installed on VUT plays an important role in the PAEB test equipment. In order to ensure the PAEB test equipment works smoothly, the communication performance and control algorithm of the cooperative control system in the PAEB test equipment is tested in open field sites. In the field tests of cooperative control performance, a remark should be made here that the AEB function of the VUT needs to be turned off, while the AEB function is turned on, when the performance of PAEB is tested for scoring its star-rating. In the field tests of cooperative control performance, both the dummy target and the VUT simultaneously reaching the predetermined collision point is used to judge the success of these tests in different test scenarios. Figure 15a shows the test scenario in which a SUVs is used as the VUT, and Figure 15b shows the base station equipment of the cooperative control system.

According to the requirement of C-NCAP (2018) for speeds of VUTs ranging from 20 to 60 km/h at 10 km/h interval, with each test being repeated three times, a total of 60 (4 × 5 × 3 = 60) trials for four different scenarios were conducted. Test results are shown in Table 6, where “1” indicates the test was successful in the first-round test conducted, and “2” indicates the test failed in the first-round test, namely the VUT and the dummy target did not arrive at the predetermined position at the first time, but successful in the second-round.

Table 6 shows that three (3) of the 20 field tests are not successful in the first-round tests, thus indicating the success rate of field tests is 85% (17/20) in the first-round tests and 100% in the second-round tests with an overall success rate of 95% (57/60). The reason for failure in the first-round tests is that the wireless communication between the dummy traction system and the ADR using 2.4 GHz communication channel was interfered by part of WLAN (wireless local area network) and Bluetooth also using the same signal channel. When field tests were conducted in an undisturbed pen filed, the success rate was significantly improved. Consequently, the tests should be conducted in an open undisturbed field, and it is best to conduct three test experiments for each test scenario to ensure and/or improve the reliability of test experiments.

## 7. Testing the Performance of the PAEB Installed in a VUT

After debugging the PAEB test equipment successfully, it can then be used to test the algorithms of AEB systems which have been embedded in their respective systems. When testing the performance of PAEB systems, the AEB function of the VUT is turned on, all other steps follow the same procedures as those previously presented in the debugging phase (see Section 5).

### 7.1. Calculating the Test Result of a PAEB System

Upon completion of the tests by the developed PAEB test equipment, the score of the AEB system installed in a VUT needs to be calculated for evaluating the performance of the AEB system. The score of the AEB system is calculated based on percentage of reduction of the VUT’s velocity between Point *V* and Point *P*_1_ (see Figure 12). Ideally, the velocity of the VUT is reduced to zero before the VUT arrives at Point *P*_1_.

The test results pertaining to the score rate and weighted value of each test speed in CVFA-50 scenario for a VUT are shown in Table 7. According to the calculation method specified in C-NCAP (2018), the scoring rate of the AEB system in CVFA-50 scenario is calculated as: (100% × 1 + 100% × 2 + 62.50% × 2 + 100% × 2 + 0% × 1)/8 = 78.12%. Then, using the same calculation method above, the scoring rates of the PAEB system in CVFA-25, CVNA-25, and CVNA-75 are 66.50%, 78.37%, and 92.60%, respectively. Hence, the comprehensive scoring rate of AEB function is (78.12% + 66.50% + 78.37% + 92.60%)/4 = 78.90%.

### 7.2. Calculating the Scoring Rate of HMI

When the preconditions of evaluating HMI are satisfied, the scoring rates of HMI are calculated as shown in Table 8.

Based on the weighted values of AEB and HMI functions from the relationship of scoring hierarchy shown in Figure 2, the comprehensive scoring rate of PAEB system is (78.90% × 5 + 100% × 1)/(5 + 1) = 82.40%, and according to the C-NCAP (2018), the score of the PAEB system is equal to 82.40% × 3 = 2.50, which is consistent with the result assessed by CATARC. The test result indicates that the developed PAEB test equipment is capable of assessing the PAEB system based on the C-NACP (2018) test requirements.

## 8. Research Results

Based upon review of the relevant literatures and analysis of the test protocol specified in C-NCAP (2018), this paper described the development of a set of PAEB test equipment and verified the test accuracy and reliability of the PAEB test equipment through either debugging or by some field experiments. The specific research results are described below.

(1) In this study, a new set of PAEB test equipment was developed including a dummy target and its traction system, an automated driving robot with precise positioning system, a monitoring device for warning performance, data operating control software, and the cooperative control system with wireless communication model. Being different from many existing publications on PAEB test equipment without disclosure of technical information, the developed test equipment was explained in minute detail in Section 3.1, from which readers would benefit in gaining a better understanding.

(2) A control method of the dummy target’s speed with S-shaped curve of acceleration and deceleration was proposed deliberately to keep the dummy target walking steadily, and the control method was simulated using MATLAB/Simulink. The simulated results demonstrated both the VUT and the dummy target were able to reach the predetermined collision point at the same time. Hence, the rationality and feasibility of the control algorithm were verified. In addition, the theoretical results can also be used as a reference for cross-checking experimental results.

(3) The control accuracy of the dummy target traction system for dummy target motion was tested in a total of 48 field tests using two SUVs, and the satisfactory rate of the dummy target traction system in PAEB test equipment was 95.83%. The test result indicated that the developed PAEB test equipment was capable of achieving accurate control of the dummy target movement in comply with the C-NACP (2018) test requirements.

(4) The communication performance and control algorithm of the cooperative control between the dummy target traction system and ADR installed on a VUT were tested in an open field, and the test results showed three of the 60 field tests were not successful in the first-round tests and 100% successful in both the second- and third-round tests with an overall success of 95%. Reasons for failure in the first-round tests was due to the wireless communication being interfered by another network also using the same signal channel. Once this problem was resolved, the test experiment of each test scenario was conducted again three rounds in an undisturbed open field, the reliability of test experiments was improved to ensure a total satisfaction.

(5) The developed PAEB test equipment was used to test the performance of the AEB installed in a VUT as presented in Section 6, and the scores of the AEB system and HMI were calculated based on the method specified in C-NCAP (2018) in accordance with evaluation schemes as presented in Section 6.1 and Section 6.2, respectively. The final scores of the PAEB system installed in a VUT was found to be 2.50, which was equal to the scores assessed by the China Automotive Technology and Research Center (CATARC), a worldwide recognized, first-class testing, research, and development center in China. Therefore, the developed PAEB test equipment can be used to test and assess the performance of the AEB system installed in a VUT based on C-NACP (2018) test protocol.

## 9. Discussion

AEB systems can effectively reduce front to rear-end accidents by 38% in real-world if the speed of vehicle is at less than 50 km/h [46], therefore they have gradually become an important vehicular active safety technology. To evaluate the performance of the AEB systems, this study presented a set of newly developed test equipment based on requirements specified in C-NCAP (2018) intended to make some contribution to the body of knowledge in active safety field. Since there are different assessment protocols specified between C-NCAP and Euro-NCAP, it is necessary to develop a set of test equipment for assessment AEB systems according to requirements specified in their respective NCAP. Examples of differences that can be mentioned are as follows: The 2018 version of Euro NCAP has added evaluations of AEB systems for cyclist detection and combined that with pedestrian detection in its 2016 version to form as AEB Vulnerable Road Users (AEB VRU) systems. In the assessment protocol specified in 2018 version of Euro NCAP, the AEB Pedestrian system is assessed in five different test scenarios including CVFA-50, CVNA-25, CVNA-75, CVNC-50 (Car-to-VRU Nearside Child 50%), CVLA-25 (Car-to-VRU Longitudinal Adult 25%), CVLA-50 (Car-to-VRU Longitudinal Adult 25%). In these test scenarios, the test speed of VUT increases from 20 km/h to 60 km/h at intervals of 5 km/h. While, in the assessment protocol specified in C-NCAP (2018), the AEB Pedestrian system is assessed in four different test scenarios including CVFA-50, CVFA-25, CVNA-25, CVNA-75; and the test speed of VUT increases from 20 km/h to 60 km/h at an intervals of 10 km/h.

During the test process, the road conditions of test fields and other communication equipment have an influence on the test results, and may cause a failed test outcome. Consequently, this study conducted these tests in an undisturbed open field which road conditions were consistent with those specified in C-NCAP. In addition, three rounds of tests in field experiments were conducted for each required test scenario to ensure the repeatability of the test equipment developed. This is recommended as a best practice when such equipment implemented in a laboratory and/or a test field.

To effectively verify the accuracy and reliability of dummy traction equipment, theoretical math-models were formulated and used to arrive at predicted results for comparison with the simulated outcomes from simulation models using MATLAB/Simulink. In addition, two SUVs equipped with AEB systems produced from two OEMs were used for testing in this study, resulting in an overall trial success of 95%. In future studies, a high-standard commercially available test equipment and the developed test equipment will be used to test the same vehicle equipped with AEB systems for comparison to further verify the accuracy of the test equipment developed and presented in this paper. In addition, it is necessary to use a 5 GHz communication band to improve the anti-interference function of the communication systems.

A couple of remarks should be made here: (1) This paper only carried out aforementioned simulations of a scenario of CVFA-50. In the future, more test scenarios will be computed and/or simulated in multiple operating modes defined in regulatory (such as Euro-NCAP and US-NCAP in addition to C-NCAP) and/or non-regulatory test protocols (i.e., IIHS for example).

(2) No other commercially available test equipment was evaluated other than the developed one presented here. This is considered as a limitation of this study. Undertaking such evaluations is crucial as a potential avenue for future studies.

## 10. Summary and Conclusions

This paper presented the research and development of a PAEB (pedestrian-AEB) test equipment system along with demonstrations of the functions of required subsystems for use in assessing a compliance of PEABs with test scenarios as specified in requirements of 2018 China New Car Assessment Program (C-NCAP). The test equipment system consisted of a dummy target and its traction system, automated driving robot (ADR), the monitoring device for FCW function, the data operating control platform, real-time communication module, and real-time kinematic (RTK). Subsystem devices adopted include not only differential GPS positioning, inertial navigation to measure the heading vector, but also ADR to precisely control the acceleration and speed of the vehicle under test (VUT) to achieve highly accurate test performance in accomplishing the objectives set forth at the beginning of this study. In addition, theoretical mathematical models using an S-shaped control algorithm were formulated for monitoring dummy target’s motion, and simulation models using MATLAB/Simulink were used to simulate various scenarios specified in C-NCAP. Furthermore, field tests on SUVs were conducted to demonstrate the developed PAEB test equipment with satisfactory results.

Conclusions can be drawn from this study as follows:

1. This PAEB test equipment was newly developed for assessing the performances of pedestrian-AEB systems in various scenarios in complying with test requirements specified in C-NCAP (2018).
2. Unlike many existing previously published studies, without providing in-depth information, this paper disclosed not only the design description of the subsystems of the developed PAEB test equipment, but also described a detailed control strategy and control method used in the PAEB test equipment developed herein.
3. An ADR in junction with differential GPS and inertial navigation device allowed achieving accurate test performance of both the VUT and the dummy target arriving at the predetermined impact location at the same time per scenario test requirement as specified in C-NCAP (2018).
4. A theoretically formulated five-stage S-shaped acceleration and deceleration control method was used for controlling the dummy target’s motion to improve the test accuracy ensuring its arrival at the predetermined impact location.
5. Simulation models based on MATLAB/Simulink were used to simulate the 30 km/h and 50 km/h test scenarios specified in the CVFA-50 (i.e., Car-to-VUR Far-side Adult-50), showing simulations yielded satisfactory results as expected.
6. Field tests of the performance of PAEB test equipment were conducted in undisturbed open 95.83%, and the success rate of the cooperative control between the dummy target traction system and the ADR installed on a VUT was 100% in the second-round tests. The test results indicated that the developed PAEB test equipment was capable of assessing the PAEB system based on the C-NACP (2018) test requirements.

A final remark is made here that the developed PAEB test equipment can provide AEB systems with means to attaining better and higher performance for future production vehicles on roads. This will play a key role in improving active safety technologies, thus impacting significantly on best practice of C-NCAP. Further, the PAEB test equipment will be upgraded under the technology of Internet-of-things, and the reliability of the sensors and the security of test equipment will be predicted by machine learning and/or artificial intelligence techniques. The PAEB test equipment when used in C-NCAP testing will also provide consumers with competent information in making their buying decisions, help manufacturers’ engineers achieve technological improvement, if any, and regulators to make potential regulatory safety decisions.

## Figures and Tables

**Table 1:** Expressions of acceleration, velocity and displacement for each stage in the S-shaped velocity curve.

Time (s)	*a*(*t*) (m/s^2^)	*v*(*t*) (m/s)	*S*(*t*) (m)
(0, *t*_1_)	*jt*	(1/2)*jt*^2^	(1/6)*jt*^3^
*t*_1_	*a_m_* = *jt*_1_	(1/2)*jt*_1_^2^	(1/6)*jt*_1_^3^
(*t*_1_, 2*t*_1_)	2*jt*_1_ − *jt*	*−jt*_1_^2^ + 2*jt*_1_*t −* (1/2)*jt*^2^	(1/3)*jt*_1_^3^ + *jt*_1_*t*^2^ − *jt*_1_^2^*t −* (1/6)*jt*^3^
*t*_2_	0	*v_t_* = *jt*_1_^2^	*jt*_1_^3^
(2*t*_1_, 2*t*_1_ + *t*_3_)	0	*v_t_* = *jt*_1_^2^	−*jt*_1_^3^ + *jt*_1_^2^*t*
(2*t*_1_ + *t*_3_, 2*t*_1_ + *t*_2_ + *t*_3_)	−*j*[*t* − 2*t*_1_ − (*t*_3_ − *t*_2_)]	*jt*_1_^2^ − (1/2) *j*[*t* − 2*t*_1_ − (*t*_3_ − *t*_2_)]^2^	(1/3)*jt*_1_^3^ + *jt*_1_*t*^2^ − *jt*_1_^2^*t* − (1/6)*jt*^3^
(2*t*_1_ + *t*_2_ + *t*_3_, 2*t*_1_ + 2*t*_2_ + *t*_3_)	−2*jt*_1_ + −*j*[*t* − 2*t*_1_ − (*t*_3_ − *t*_2_)]	(1/2) *j*[*t* − 4*t*_1_ − (*t*_3_ − *t*_2_)]^2^	−(26/3)*jt*_1_^3^ + 8 *jt*_1_^2^*t*−2*jt*_1_*t*^2^ + (1/6)*jt*^3^

**Table 2:** The detail data of the simulation results in S-shaped velocity curve.

Section	Running State	*v*(*t*) (m/s)	*a*(*t*) (m/s^2^)	Time (s)	*j* (m/s^3^)
[0, *T*_1_]	Increasing acceleration	$v_{t_{1}}=(1/2)j\cdot t_{1}^{2}$ = 0.90	1.80	1.00	1.80
[*T*_1_, *T*_2_]	Decreasing acceleration	$v_{t_{2}}=v_{t}=j\cdot t_{1}^{2}$ = 1.80	0.00	1.00	−1.80
[*T*_2_, *T*_3_]	Uniform velocity	$v_{t_{3}}=v_{t}=j\cdot t_{1}^{2}$ = 1.80	0.00	*T*_3_	0
[*T*_3_, *T*_4_]	Increasing deceleration	$v_{t_{4}}=(1/2)j\cdot t_{1}^{2}$ = 0.90	−1.80	1.00	−1.80
[*T*_4_, *T*_5_]	Decreasing deceleration	$v_{t_{5}}=0.00$	0.00	1.00	1.80

**Table 3:** The longitudinal coordinate values of Point M and vehicle center Point G at each test velocity.

Items	The Velocity of VUT (km/h)				
	20	30	40	50	60
*y*_3_ of point *M*	28.75	43.07	57.44	71.81	86.18
*y*_5_ of point *G*	31.00	45.32	59.69	74.06	88.43

**Table 4:** Test of the walking distance and speed of the dummy target (with SUV1).

Test Scenario		Distance (m)				Velocity (km/h)				
	Test Results			Standard Value	Valid Range	Test Results			Standard Value	Valid Range
CVFA-25	5.53	5.50	5.49	5.52	5.49–5.55	6.50	6.55	6.50	6.5	6.3–6.7
CVFA-50	6.02.	**5.96**	6.00	6.00	5.97–6.03	6.52	6.48	6.50	6.5	6.3–6.7
CVNA-25	**3.60**	3.52	3.53	3.52	3.49–3.55	5.10	4.96	4.98	5.0	4.8–5.2
CVNA-75	4.50	4.45	4.50	4.48	4.45–4.51	5.06	4.94	5.10	5.0	4.8–5.2

Note: Every test scenario is repeatedly tested three times.

**Table 5:** Test of the walking distance and speed of the dummy target (with SUV2).

Test Scenario		Distance (m)				Velocity (km/h)				
	Test Results			Standard Value	Valid Range	Test Results			Standard Value	Valid Range
CVFA-25	5.50	5.52	5.48	5.51	5.48–5.54	6.52	6.60	6.56	6.5	6.3–6.7
CVFA-50	5.98	6.02	6.00	6.00	5.97–6.03	6.50	6.54	6.52	6.5	6.3–6.7
CVNA-25	3.54	3.50	3.52	3.51	3.48–3.54	5.18	5.06	5.10	5.0	4.8–5.2
CVNA-75	4.50	4.52	4.48	4.49	4.46–4.52	5.08	5.12	5.06	5.0	4.8–5.2

Note: Every test scenario is repeatedly tested three times.

**Table 6:** Testing the cooperative control between the dummy traction system and ADR.

Velocity (km/h)	Test Scenarios in C-NCAP (2018)			
	CVFA-25	CVFA-50	CVNA-25	CVNA-75
20	1	1	1	1
30	1	1	1	2
40	1	1	1	1
50	1	1	2	1
60	2	1	1	1

**Table 7:** The test results at each test velocity in CVFA-50 scenario.

*v*_test_ in km/h	*v*_impact_ in km/h	Scoring Rate	Weighted Value
20	0	100.00%	1
30	0	100.00%	2
40	15	62.50%	2
50	30	100.00%	2
60	46	0.00%	1

**Table 8:** The score in evaluation of HMI.

Items	Weighted Value	Score	Scoring Rate
Closing request	2	2.00	66.78%
FCW alarm	1	1.00	33.33%
HMI	3	3.00	100.00%

## Abbreviations

ADR	Automated driving robot
ASS	Active safety system
CATARC	China Automotive Technology and Research Center
CATS	Cyclist-AEB Testing System
C-NCAP	China-new car assessment program
CVFA-50	Car-to-VRU Farside Adult-50
CVFA-25	Car-to-VRU Farside Adult-25
CVNA-25	Car-to-VRU Nearside Adult-75
CVNA-75	Car-to-VRU Nearside Adult-75
CCR	Car-to-car rear collision
CCRb	Car to car rear braking
CCRm	Car to car rear moving
CCRs	Car to car rear stationary
DC	Direct-current
DfT	Department for Transportation in UK
DOF	Degrees of freedom
DGPS	Differential global positioning system
ECU	Electronic control unit
Euro NCAP	European new car assessment program
FCW	Forward collision warning
GM	General Motors
GPS-RTK	Global positioning system- real-time kinematic
GPS	Global positioning system
HMI	Human-machine interface
INS	Inertial navigation system
MFC	Microsoft Foundation Classes
NMEA	National Marine Electronics Association
NCAP	New car assessment program
NHTSA	National Highway Transportation Safety Administration
OEMs	Original equipment manufacturers
OTS	On-the-spot
PAEB	Pedestrian-automatic emergency braking
PSS	Passive safety system
PDL	Positioning data link
PROSPECT	Proactive safety for pedestrians and cyclists
RTK	Real-time kinematic
TTC	Time to collision
VT	Vehicle target
VRUs	Vulnerable road users
VUT	Vehicle under test
WHO	World Health Organization
WLAN	Wireless local area network
